# Disrupted Brain Functional Network Architecture in Chronic Tinnitus Patients

**DOI:** 10.3389/fnagi.2016.00174

**Published:** 2016-07-08

**Authors:** Yu-Chen Chen, Yuan Feng, Jin-Jing Xu, Cun-Nan Mao, Wenqing Xia, Jun Ren, Xindao Yin

**Affiliations:** ^1^Department of Radiology, Nanjing First Hospital, Nanjing Medical UniversityNanjing, China; ^2^Department of Otolaryngology, Nanjing First Hospital, Nanjing Medical UniversityNanjing, China; ^3^Department of Endocrinology, Nanjing First Hospital, Nanjing Medical UniversityNanjing, China

**Keywords:** chronic tinnitus, degree centrality, effective connectivity, resting-state fMRI

## Abstract

**Purpose**: Resting-state functional magnetic resonance imaging (fMRI) studies have demonstrated the disruptions of multiple brain networks in tinnitus patients. Nonetheless, several studies found no differences in network processing between tinnitus patients and healthy controls (HCs). Its neural bases are poorly understood. To identify aberrant brain network architecture involved in chronic tinnitus, we compared the resting-state fMRI (rs-fMRI) patterns of tinnitus patients and HCs.

**Materials and Methods**: Chronic tinnitus patients (*n* = 24) with normal hearing thresholds and age-, sex-, education- and hearing threshold-matched HCs (*n* = 22) participated in the current study and underwent the rs-fMRI scanning. We used degree centrality (DC) to investigate functional connectivity (FC) strength of the whole-brain network and Granger causality to analyze effective connectivity in order to explore directional aspects involved in tinnitus.

**Results**: Compared to HCs, we found significantly increased network centrality in bilateral superior frontal gyrus (SFG). Unidirectionally, the left SFG revealed increased effective connectivity to the left middle orbitofrontal cortex (OFC), left posterior lobe of cerebellum (PLC), left postcentral gyrus, and right middle occipital gyrus (MOG) while the right SFG exhibited enhanced effective connectivity to the right supplementary motor area (SMA). In addition, the effective connectivity from the bilateral SFG to the OFC and SMA showed positive correlations with tinnitus distress.

**Conclusions**: Rs-fMRI provides a new and novel method for identifying aberrant brain network architecture. Chronic tinnitus patients have disrupted FC strength and causal connectivity mostly in non-auditory regions, especially the prefrontal cortex (PFC). The current findings will provide a new perspective for understanding the neuropathophysiological mechanisms in chronic tinnitus.

## Introduction

Tinnitus is the perception of a sound in the absence of an external sound source. Roughly 12% of adults experience tinnitus, but the prevalence skyrockets to 50% in combat personnel (McFadden, 1982; Meikle, 1997). Chronic tinnitus usually leads to problems ranging from mild discomfort such as sleep disturbance to strong anxiety and depression (Leske, 1981; Lockwood et al., 2002). Although most tinnitus patients localize tinnitus to one or both ears, the severity of the phantom sound could not be eliminated after sectioning the auditory nerve (Berliner et al., 1992; Jackler and Whinney, 2001). Tinnitus patients show various pathophysiological changes, such as increased spontaneous activity, enhanced neural synchrony, tonotopic map reorganization, abnormal consciousness gating mechanisms and coupling of networks involving auditory and non-auditory structures (Lenarz et al., 1993; Lockwood et al., 1998; Kaltenbach et al., 2005; Adjamian et al., 2009; Henry et al., 2014). Nonetheless, the mechanisms that maintain the disorder remain poorly understood.

Previous neuroimaging studies using electroencephalography (EEG), magnetoencephalography (MEG), positron emission tomography (PET), and functional magnetic resonance imaging (fMRI), have investigated the neuropathophysiological mechanisms implicated in chronic tinnitus (Shulman and Strashun, 1998; Mirz et al., 1999; Adjamian et al., 2009; Schlee et al., 2009; Roberts et al., 2010; Vanneste and De Ridder, 2012). Resting-state fMRI (rs-fMRI) is a promising noninvasive technique that could reflect the brain functional architecture in low frequency fluctuations (0.01–0.1 Hz) of blood oxygenation level-dependent (BOLD; Biswal et al., 1995; Fox and Raichle, 2007). Using rs-fMRI, multiple brain networks relevant to neural mechanisms of tinnitus have been demonstrated, such as the auditory network (Burton et al., 2012; Kim et al., 2012; Maudoux et al., 2012a,b; Schmidt et al., 2013; Hinkley et al., 2015; Minami et al., 2015; Leaver et al., 2016), dorsal attention network (DAN; Burton et al., 2012; Schmidt et al., 2013), ventral attention network (VAN; Burton et al., 2012), default mode network (DMN; Schmidt et al., 2013; Chen et al., 2014, 2015d; Leaver et al., 2016), and visual network (Burton et al., 2012; Chen et al., 2014). However, these results have been variable due to applied analytic methods. Burton et al. (2012) implied that there is dissociation between activity in auditory cortex and visual, attention and control networks using seed-based functional connectivity (FC) analysis. Through independent component analysis (ICA), Kim et al. (2012) found enhanced FC between the attention network and auditory network, suggesting that this network might contribute to the perception or salience of tinnitus. Schmidt et al. (2013) identified specific alterations in the connectivity of the DMN, DAN, and auditory networks due to tinnitus. Similarly, Maudoux et al. (2012a,b) provided fMRI evidence for a distributed network of auditory and non-auditory cortical and sub-cortical regions associated with chronic tinnitus pathology using ICA approach. Using the algorithms of amplitude of low-frequency fluctuations (ALFF) and regional homogeneity (ReHo), Chen et al. (2014, 2015c,d) found abnormal spontaneous neural activity within multiple cerebral networks, such as the DMN and the attention network. Nonetheless, other studies have failed to detect any differences in network processing between tinnitus patients and controls (Wineland et al., 2012; Davies et al., 2014). Heterogeneity among tinnitus cohorts may contribute to network state variations. According to these recent theories and observations, we speculated that abnormal brain FC and network might underlie the pathophysiology in chronic tinnitus.

The prefrontal cortex (PFC) exerts early inhibitory modulation of input to primary auditory cortex in humans and has been found to be associated with auditory attention (Lewis et al., 2000; Voisin et al., 2006). The vital role for the PFC in subserving tinnitus mechanism has been postulated (Jastreboff, 1990) and previous neuroimaging studies have confirmed the involvement of PFC for tinnitus (Vanneste et al., 2010, 2012; Vanneste and De Ridder, 2012; De Ridder et al., 2014). Furthermore, latest rs-fMRI studies identified abnormalities in the PFC associated with tinnitus (Burton et al., 2012; Kim et al., 2012; Schmidt et al., 2013; Ueyama et al., 2013; Chen et al., 2014, 2015b,c,d; Leaver et al., 2016). Among them, Ueyama et al. (2013) observed disrupted FC strength in distinct brain regions, especially in the left superior frontal gyrus (SFG), which was negatively correlated with tinnitus loudness. Taken together, the PFC is considered as a key region involved in tinnitus. Investigations exploring the role of PFC in the brain functional network architecture of tinnitus will provide valuable insight into the neural mechanisms underlying the chronic tinnitus.

Seed-based FC and ICA approaches have proved extremely useful in exploring connectivity patterns for specific components of interest. However, few studies have investigated the tinnitus-related alterations of whole-brain FC pattern or large-scale brain network. Degree centrality (DC) is a voxel-wise data-driven method that can quantify the importance of each node in brain network. This graph theory based network analysis can assess the network centrality without* a priori* selection of nodes or networks of interest (Zuo et al., 2012). This algorithm has been used to observe the alterations of resting-state functional networks in diverse diseases, such as Alzheimer’s disease (AD), autism, and hepatic encephalopathy (Buckner et al., 2009; Di Martino et al., 2013; Chen et al., 2015a). Since the neural mechanisms underlying tinnitus are poorly understood and multiple brain systems are involved, we applied DC to analyze FC within the whole-brain network. To examine the directional connectivity network involved in tinnitus, we further used the granger causality analysis (GCA), which is a statistical method originally used in the field of economics to assess directional influences between simultaneously recorded time series (Granger, 1969; Zhou et al., 2011). GCA has been widely used to reveal the causal effects among brain regions in various neurological or psychiatric disorders, such as AD, schizophrenia, depression and hepatic encephalopathy (Qi et al., 2013; Zhong et al., 2014; Guo et al., 2015a,b). Thus, to unravel the details of the brain functional network architecture in tinnitus, we sought to evaluate the brain regions that show aberrant FC across the entire brain networks in tinnitus patients using DC and then use GCA to analyze effective connectivity to understand the directional aspect of these alterations. We hypothesized that the intrinsic dysconnectivity pattern of the PFC might play a crucial role in the brain functional network architecture of tinnitus patients.

## Materials and Methods

### Subjects

All the subjects provided written informed consent before their participation in the study protocol, which was approved by the Research Ethics Committee of the Nanjing Medical University (Reference No. 2016067).

According to both the inclusion and exclusion criteria of this study, a final sample of 47 subjects including 25 chronic tinnitus patients and 22 healthy controls (HCs) were recruited through community health screening and newspaper advertisements. The tinnitus patients and healthy subjects were group-matched in terms of age, sex, and education. One tinnitus patient was subsequently excluded because the limits for head motion were exceeded during MR scanning. Ten patients reported a predominantly left-sided tinnitus, six a predominantly right-sided tinnitus and eight patients described their tinnitus as bilateral or originating within the head. All subjects were right-handed and completed at least 8 years of education. The severity of tinnitus and related distress were assessed by the Iowa version of the Tinnitus Handicap Questionnaires (THQ; Kuk et al., 1990). Hearing thresholds were determined by puretone audiometry (PTA). All of the participants had normal hearing (defined as thresholds <25 dB HL) at the frequencies of 0.25 kHz, 0.5 kHz, 1 kHz, 2 kHz, 4 kHz, and 8 kHz. There were no significant differences in auditory thresholds between tinnitus and control groups. In addition, none of the participants had symptoms of depression and anxiety according to the Self-Rating Depression Scale (SDS) and Self-Rating Anxiety Scale (SAS; overall scores <50, respectively; Zung, 1971, 1986). According to previous study (Khalfa et al., 2002), we used the Hyperacusis Questionnaire to exclude the participants with hyperacusis in the current study. Participants were also excluded from the study if they suffered from pulsatile tinnitus or Meniere’s diseases, or if they had a past history of severe smoking, stroke, alcoholism, brain injury, Parkinson’s disease, AD, epilepsy, major depression, neurological or psychiatric disorders that could affect cognitive function, major medical illness (e.g., anemia, thyroid dysfunction and cancer), MRI contraindications (e.g., cochlear implants, pacemakers, cerebral aneurysm clips, prosthetic valves, a history of intraocular metal fragments, and claustrophobia), or severe visual loss. The characteristics of the chronic tinnitus patients and healthy subjects are summarized in Table 1.

**Table 1:** **Characteristics of the tinnitus patients and healthy controls (HCs)**.

	Tinnitus patients (*n* = 24)	HCs (*n* = 22)	*p* value
Age (year)	50.8 ± 12.4 (26–67)	44.7 ± 15.4 (26–70)	0.144
Gender (male: female)	9:15	9:13	0.813
Education levels (years)	12.3 ± 3.1 (8–18)	13.4 ± 3.8 (8–22)	0.313
Tinnitus duration (months)	46.5 ± 39.1 (6–120)	–	–
THQ score	49.5 ± 15.5	–	–
Hearing thresholds (left)	13.0 ± 2.7	13.6 ± 2.2	0.414
Hearing thresholds (right)	14.6 ± 3.6	13.9 ± 3.4	0.470

*Data are represented as Mean ± SD (range of min-max)*.

### MRI Acquisition

MRI data were acquired at our hospital using a 3.0 T MRI scanner (Ingenia, Philips Medical Systems, Netherlands). Head motion and scanner noise were reduced using foam padding and earplugs. The earplugs (Hearos Ultimate Softness Series, USA) were used to attenuate scanner noise by approximately 32 dB. Subjects were instructed to lie quietly with their eyes closed without falling asleep, not think of anything in particular, and avoid any head motion during the scan. Functional images were obtained axially using a gradient echo-planar imaging (EPI) sequence as follows: repetition time (TR) = 2000 ms; echo time (TE) = 30 ms; slices = 36; thickness = 4 mm; gap = 0 mm; field of view (FOV) = 240 mm × 240 mm; acquisition matrix = 64 × 64; and flip angle (FA) = 90°. The fMRI sequence took 8 min and 8 s. Structural images were acquired with a three-dimensional turbo fast echo (3D-TFE) T1WI sequence with high resolution as follows: TR/TE = 8.1/3.7 ms; slices = 170; thickness = 1 mm; gap = 0 mm; FA = 8°; acquisition matrix = 256 × 256; FOV = 256 mm × 256 mm. The structural sequence took 5 min and 29 s.

### Functional Data Preprocessing

Functional data analyses were conducted using Data Processing Assistant for Resting-State fMRI (DPARSF) programs (Chao-Gan and Yu-Feng, 2010) based on statistical parametric mapping (SPM8^1^) and rs-fMRI data analyses toolkits (REST^2^). A total of 240 volumes were scanned, and the first 10 volumes were discarded to allow for signal equilibrium of the initial magnetic resonance signals and adaptation of the subjects to scanner. The remaining 230 consecutive volumes were used for data analysis. Afterwards, the following procedures were carried out as follows: slice-timing adjustment, realignment for head-motion correction, spatial normalization to the Montreal Neurological Institute (MNI) template (resampling voxel size = 3 × 3 × 3 mm^3^) and smoothing with an isotropic Gaussian kernel (full width at half maximum (FWHM) = 6 mm), detrending and filtering (0.01–0.08 Hz). Any subjects with a head motion >2.0 mm translation or a 2.0° rotation in any direction were excluded.

### Degree Centrality Analysis

We restricted our voxel-wise centrality analyses to a predefined gray matter (GM) mask that included tissue with GM probabilities greater than 20% as previously described (Zuo et al., 2012). Within the mask, individual network centrality maps were generated in a voxel-wise fashion. First, the preprocessed functional runs were subjected to voxel-based whole-brain correlation analysis. The time course of each voxel from each participant was correlated with the time course of every other voxel, which resulted in a correlation matrix. An undirected adjacency matrix was then obtained by thresholding each correlation at *r* > 0.25 (Buckner et al., 2009; Zuo et al., 2012; Yan et al., 2013). As previously reported, the negative correlations were not included in DC calculation, given their ambiguous interpretation and detrimental effects on test-retest reliability (Buckner et al., 2008; Vincent et al., 2008; Murphy et al., 2009). A high threshold was chosen to eliminate counting voxels that had low temporal correlation attributable to signal noise. Different threshold selections did not qualitatively change the results for cortex (Buckner et al., 2009). Then, the DC was computed as the number of significant correlations (binarized) or as the sum of the weights of the significant connections (weighted) for each voxel (Buckner et al., 2009; Zuo et al., 2012). This measure of connectivity (degree, *D*) for each voxel (*i*) with all other voxels (*j*) is given by the following: *D_i_* = ∑*d_ij_* where, *j* = 1 … *N*, *i* ≠ *j*. The map of the connectivity was then standardized by converting to *z* scores so that maps across participants could be averaged and compared. The *z* score transformation is given by:

(1) $$Z_{i}=\frac{D_{i}-\bar{D}}{\sigma_{D}}\;i=1\ldots N$$

The $\bar{D}$ is the mean degree across all the voxels in the whole-brain map and *σ_D_* is the standard deviation of the map. DC has been shown to represent the most local and directly quantifiable centrality measure and has been widely used to examine node characteristics of intrinsic network connectivity (Zuo et al., 2012). The DC maps were transferred to *z*-values for group comparisons. Within brain network, the DC value of a node indicates its connectivity strength to all the other nodes and reflects its importance in functional integration.

We first estimated spatial distribution of mean DC in the tinnitus group and healthy group, respectively. The individual *z* values were entered into the SPM8 software for a random effect one-sample *t*-test in a voxel-wise way to show the average DC maps within each group. The significant threshold was set at *p* < 0.01, with multiple comparisons correction using the AFNI AlphaSim program^3^ determined by Monte Carlo simulation (AlphaSim program with following parameters: single voxel *p* value of 0.01, a minimum cluster size of 40 voxels, 5000 simulations, cluster connection radius *r* = 5 mm, FWHM = 6 mm). This correction was confined within the aforementioned GM mask.

To find the disrupted brain hub regions, two-tailed two-sample *t*-test were then conducted to investigate the differences in the DC maps between tinnitus patients and HCs. Between-group comparisons of the DC maps were performed in the SPM8 software using general linear model (GLM) analysis, with age, sex and education included as nuisance covariates. A correction for multiple comparisons was performed by a Monte Carlo simulation using the AlphaSim program, resulting in a corrected threshold of *p* < 0.01 and minimum cluster size of 40 voxels (parameters were single voxel *p* value of 0.01, 5000 simulations, cluster connection radius *r* = 5 mm, FWHM = 6 mm). For between-group analysis, a mask was created by combining the significant clusters in both groups, which were obtained from one-sample *t*-test results.

### Effective Connectivity Analysis

Using DC approach, we were able to show that the bilateral SFG is a region of special functional importance in tinnitus patients revealing increased FC. To further investigate the influence of directionality, we applied GCA to evaluate changes in effective connectivity. Based on the results of the DC analysis we selected the seed regions, which showed significant differences between tinnitus patients and HCs (left and right SFG: MNI coordinates (*x, y, z*) ±18, 42, 27). Effective connectivity was analyzed using REST-GCA in the REST toolbox (Zang et al., 2012). In this study, two separate time series of the left and right SFG were defined as the seed time series *x*, and the time series *y* denotes the time series of all voxels in the brain. The linear direct influence of *x* on *y* (F_*x→y*_), and the linear direct influence of *y* on *x* (F_*y→x*_) were calculated voxel by voxel across the brain. Thus, two Granger causality maps were generated based on the influence measures for each subjects. The residual-based F was normalized (F′) and standardized to Z score for each voxel (Z_*x→y*_ and Z_*y→x*_, subtracting the global mean F′values, divided by standard deviation).

For the group analysis on the effective connectivity, mean values of Z_*x→y*_ and Z_*y→x*_ maps were computed for each group. All eight Granger causality maps were acquired, with four for each direction and four for each group (the left SFG with Z_*x→y*_ and Z_*y→x*_ and the right SFG with Z_*x→y*_ and Z_*y→x*_ for both tinnitus patient and HCs). Then these Granger causality maps were entered into SPM8 software for group comparison. A random effect two-sample *t*-test in a voxel-wise manner was performed to determine the differences of effective connectivity of the SFG between tinnitus patient and HCs, with age, sex and education including as nuisance covariates. A correction for multiple comparisons was also conducted by a Monte Carlo simulation using the AlphaSim program, with a corrected threshold of *p* < 0.01 and minimum cluster size of 40 voxels.

### Correlation Analysis

To investigate the relationship between clinical characteristic of tinnitus patients and DC and effective connectivity measures, these regions showing significant differences in DC or effective connectivity between groups were extracted. Mean *z* values within these clusters were correlated against each tinnitus characteristic using the Pearson’s correlation analysis by SPSS software (version 18.0; SPSS, Chicago, IL, USA). *P* < 0.05 was considered statistically significant, corrected for age, sex and education. Bonferroni correction for multiple comparisons was applied in the correlation analysis.

## Results

### Degree Centrality Analysis

**Figure 1:**
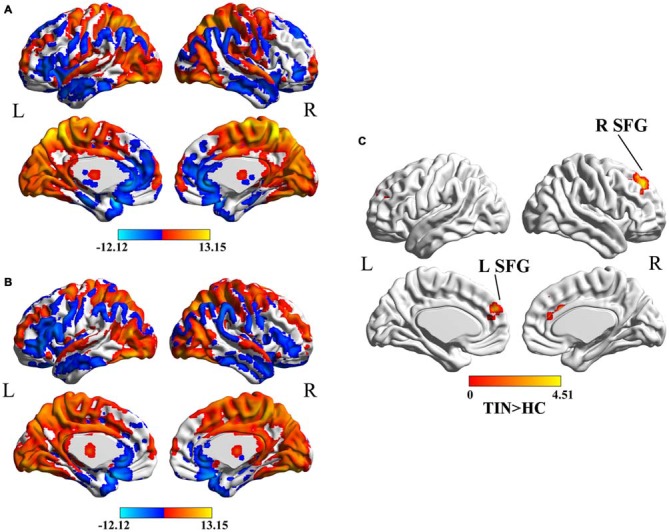
**(A)** Spatial distribution of the degree centrality (DC) in the healthy controls (HCs). **(B)** Spatial distribution of the DC in the chronic tinnitus patients. **(C)** Significantly increased DC within the bilateral superior frontal gyrus (SFG) between tinnitus patients and HCs. TIN, tinnitus; HCs, healthy controls.

In both HC (Figure 1A) and tinnitus patients (Figure 1B), the spatial distribution of the weighted DC was highly localized in the occipital lobe, inferior parietal lobe (IPL), PFC, cingulate cortex, insula and thalamus. After two-sample *t*-test analysis, significantly increased DC within the bilateral SFG was found in tinnitus patients compared to HCs (*p* < 0.01, AlphaSim corrected; Figure 1C).

### Effective Connectivity Analysis

**Figure 2:**
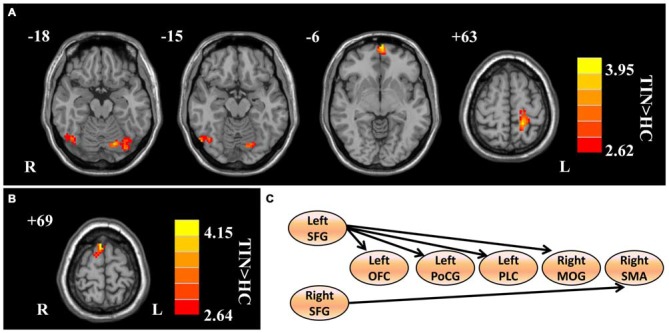
**Aberrant effective connectivity from the bilateral SFG in tinnitus patients. (A)** Increased effective connectivity from the left SFG to the left orbitofrontal cortex (OFC), left PoCG, left posterior lobe of cerebellum (PLC), and right middle occipital gyrus (MOG; *p* < 0.01, AlphaSim corrected). **(B)** Increased effective connectivity from the right SFG to the right supplementary motor area (SMA; *p* < 0.01, AlphaSim corrected). **(C)** Schematic overview of changes in effective connectivity from the bilateral SFG. SFG, superior frontal gyrus; OFC, orbitofrontal cortex; PoCG, precentral gyrus; PLC, posterior lobe of cerebellum; MOG, middle occipital gyrus; SMA, supplementary motor area; TIN, tinnitus; HCs, healthy controls.

Compared to HCs, patients with chronic tinnitus demonstrated significantly increased effective connectivity from the left SFG to the left middle orbitofrontal cortex (OFC), left posterior lobe of cerebellum (PLC), left postcentral gyrus (PoCG), and right middle occipital gyrus (MOG). Moreover, the right SFG exhibited enhanced effective connectivity to the right supplementary motor area (SMA; *p* < 0.01, AlphaSim corrected; Figure 2 and Table 2). However, we did not find any abnormal feedback effect to the bilateral SFG in the tinnitus patients.

**Table 2:** **Regions showing significant differences in effective connectivity between tinnitus patients and HCs**.

Brain regions	BA	Peak MNI coordinates *x, y, z* (mm)	Peak *T* value	Voxels

**Increased effective connectivity from left SFG**				
Left OFC	11	−3, 66, −6	4.1302	158
Left PoCG	3	−18, −42, 63	4.0470	132
Left PLC	–	−18, −75, −18	3.7193	100
Right MOG	19	54, −69, −15	3.6185	46
**Increased effective connectivity from right SFG**				
Right SMA	6	6, 15, 69	4.4562	44

*A corrected threshold of p < 0.01 was determined by Monte Carlo simulation. BA, Brodmann’s area; MNI, Montreal Neurological Institute; SFG, superior frontal gyrus; OFC, orbitofrontal cortex; PoCG, precentral gyrus; PLC, posterior lobe of cerebellum; MOG, middle occipital gyrus; SMA, supplementary motor area*.

### Correlation Results

**Figure 3:**
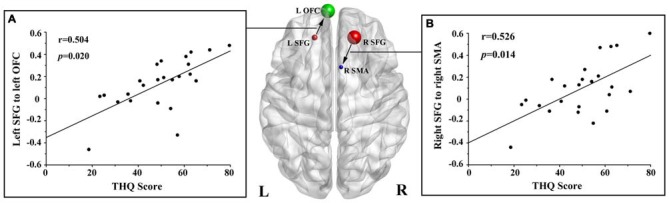
**Correlations between the increased effective connectivity of the bilateral SFG and the tinnitus handicap questionnaires (THQ) scores. (A)** The THQ scores positively correlated with the increased effective connectivity from the left SFG to left OFC (*r* = 0.504, *p* = 0.020). **(B)** The THQ scores positively correlated with the increased effective connectivity from the right SFG to right SMA (*r* = 0.526, *p* = 0.014). L, left; R, right; THQ, tinnitus handicap questionnaires; SFG, superior frontal gyrus; OFC, orbitofrontal cortex; SMA, supplementary motor area.

We found no significant correlations between the DC of the bilateral SFG and the tinnitus characteristics. However, the THQ scores positively correlated with the increased effective connectivity from the left SFG to left OFC (*r* = 0.504, *p* = 0.020), and from the right SFG to right SMA (*r* = 0.526, *p* = 0.014; Figure 3). The other regions with enhanced effective connectivity revealed no significant correlations with tinnitus duration or THQ scores.

## Discussion

This is the first study to use both DC and GCA approaches to explore intrinsic functional network architecture related to tinnitus. Using DC analysis, we found significantly increased network centrality within the bilateral SFG regions in chronic tinnitus patients. Using GCA algorithm, we employed the bilateral SFG as seeds to examine their causal effect with the whole brain. Unidirectionally, bilateral SFG showed increased effective connectivity to several non-auditory regions including the prefrontal, motor, visual cortex and cerebellum. Of note, the abnormal connectivity to the OFC and the SMA revealed significant correlations with the tinnitus distress.

### Increased Network Centrality in the SFG

Certain regions show strong connections with other regions within large-scale cortical networks that constitute an emerging feature of brain architecture (Sporns et al., 2007; Buckner et al., 2009; Zuo et al., 2012). Our findings show that the SFG is the main cortical hub in the brain network architecture affected by tinnitus, which is in line with the hypothesis that the dysconnectivity pattern of the PFC involved in tinnitus perception. The PFC has been regarded as a critical region by Jastreboff (1990), who suggested that the PFC integrates sensory and emotional aspects of tinnitus. Several neurophysiological models based on neuroimaging have been raised involved in tinnitus subsequently (De Ridder et al., 2014). Rauschecker et al. (2010) developed a model to demonstrate structural and functional differences in ventromedial prefrontal cortex (vmPFC) that were associated with tinnitus subjective loudness, indicating that PFC may contribute to certain perceptual features of tinnitus (Leaver et al., 2011). The current integrative model attempts to unify the different brain areas and networks associated with tinnitus in one model and propose the hypothetical neural core of conscious phantom sound perception (De Ridder et al., 2011, 2014; Vanneste and De Ridder, 2012), which is more extensive than the initially proposed auditory system (Jastreboff and Hazell, 2008).

Our current study suggested that the PFC, specifically the SFG, might be a major integrative hub of the model that should be involved to perceive tinnitus. However, we did not observe any abnormal neural activity in auditory system. The most parsimonious explanation for this is the absence of any hearing loss out to 8 kHz and the absence of hyperacusis in our tinnitus patients. We speculated that aberrant neural activity of the PFC may exist prior to the disruption of the auditory system in tinnitus patients with normal hearing. In addition, since our tinnitus patients showed no obvious symptoms of depression, anxiety or cognitive decline, we did not detect any abnormalities in the limbic system. Based on previous fMRI studies, the SFG is a significant part of auditory connection cortex that can receive and integrate all kinds of information from different parts of the brain from inside and outside the body. Besides, it can also timely organize efferent impulses to ensure the coordination of the central nervous system (CNS) as a whole (Mathiak et al., 2007; Melloni et al., 2007). A possible interpretation for our results was that the increased DC in bilateral SFG might be due to feedback inhibition of the over activity in auditory network.

Previous neuroimaging studies have pointed out that the abnormalities of the SFG could act as a direct mechanism of tinnitus chronification (Giraud et al., 1999; Mirz et al., 1999; Haller et al., 2010). Wunderlich et al. (2010) detected the SFG activation after acoustic stimulation in a pitch discrimination task, suggesting the perception of auditory inputs in a more emotional context due to tinnitus. Chen et al. (2014) found increased ALFF values in the right SFG in chronic tinnitus patients, which was linked with tinnitus duration and distress. The SFG was also proved to be influenced by chronic tinnitus in recent rs-fMRI studies (Maudoux et al., 2012a; Ueyama et al., 2013; Chen et al., 2015c; Zhang et al., 2015). In line with these valuable findings, our results have implications for understanding the specific role of SFG abnormalities in chronic tinnitus, suggesting the PFC may provide valuable insight into the neural mechanisms underlying tinnitus.

### Increased Effective Connectivity from the SFG

Using a GCA method, the present study exhibited that the information flow is unidirectionally affected (seed to whole brain) within non-auditory areas due to tinnitus. We found increased influence from the SFG to the frontal cortex (OFC and SMA), cerebellum (PLC), visual cortex (MOG) and somatosensory cortex (PoCG). Previous neuroimaging studies have identified abnormalities in the frontal cortex which could account for tinnitus. Firstly, it has been revealed that the OFC is critical for emotional processing of sounds (Damasio et al., 1996; Blood et al., 1999). The alterations of the OFC have been found in the functional coupling of long-range cortical networks between tinnitus patients and HCs by using EEG or MEG (Schlee et al., 2009; Vanneste et al., 2012; Song et al., 2014). Moreover, recent rs-fMRI studies have also demonstrated the abnormal FC of the OFC in tinnitus patients (Maudoux et al., 2012a; Chen et al., 2015d; Zhang et al., 2015). The OFC is also regarded as part of the reward system, which might integrate the aversive information of the perceived tinnitus (Rolls, 2004; Kringelbach, 2005). The heightened effective connectivity from the SFG to the OFC might be interpreted as a dysfunctional inhibitory response directing attention away from phantom sound perception. Our finding of positive correlation between the effective connection from the SFG to the OFC and tinnitus distress also emphasized the pivotal role of the OFC in tinnitus. Furthermore, we also found positive correlation between the THQ score and increased influence from the SFG to the SMA, which is regarded as a part of the primate cerebral cortex that contributes to the control of movement (Roland et al., 1980). By using quantitative EEG (qEEG), Vanneste et al. (2011) observed aberrant neuronal activity in the SMA in unilateral or bilateral tinnitus (Vanneste and De Ridder, 2012). It was hypothesized that synchronized theta activity in the SMA might be accountable for part of the conscious perception of the phantom sound (Vanneste and De Ridder, 2012). Nevertheless, further research is needed to clarify the role of the SMA in tinnitus.

Although the cerebellum is primarily involved in motor actions and control, some cerebellar regions such as the paraflocculus and vermis receive inputs from auditory centers (Petacchi et al., 2005). Auditory sensory processing in the cerebellum has been reported. Osaki et al. (2005) revealed that the right cerebellum was involved in tinnitus and showed a decreased regional blood flow during residual inhibition. Using rs-fMRI, chronic tinnitus patients showed increased FC in the cerebellar hemisphere that was linked with tinnitus distress (Maudoux et al., 2012a; Ueyama et al., 2013), confirming the involvement of cerebellum in auditory system. Nevertheless, the association of the cerebellum with tinnitus has not been substantially elucidated. Moreover, increased effective connectivity to MOG and PoCG raised the question of whether tinnitus might be linked to phantom visual or somatosensory perceptions. Such changes seem reasonable given the multisensory interactions known to exist between auditory, visual and somatosensory regions. Possible neural correlates of visual or somatosensory modulation of tinnitus were assessed (Murray et al., 2005). One interpretation of these results is that as patients attend to their phantom auditory sensation they contemporaneously activate visual or somatosensory areas. This interpretation is consistent with previous rs-fMRI studies showing abnormal neural activity in visual network (Burton et al., 2012; Maudoux et al., 2012a; Chen et al., 2014, 2015b,c,d) or somatosensory network (Maudoux et al., 2012a; Ueyama et al., 2013) in tinnitus. Therefore, tinnitus can be regarded as the consequence of multiple brain subnetworks involved in the different aspects of tinnitus, both acoustic and affective.

### Tinnitus Lateralization

Interestingly, results of effective connectivity analysis were lateralized to the left hemisphere region in tinnitus patients. Asymmetry for the tinnitus patients has been reported both structurally and functionally (Mühlau et al., 2006; Smits et al., 2007; Schecklmann et al., 2013; Chen et al., 2014). Previous PET studies have demonstrated an overactivation of the left auditory cortex independent of tinnitus laterality and anatomical hemispheric differences (Arnold et al., 1996; Langguth et al., 2006; Schecklmann et al., 2013). This lateralization may be explained as an increase in activation on the side of the perceived tinnitus, or a decrease in activation on the side contralateral to the side of perceived tinnitus. The interpretation would indicate an increased spontaneous neural activity of the affected brain area in tinnitus patients (Smits et al., 2007). However, several studies also confirmed the right-lateralization in tinnitus (Chen et al., 2014; Geven et al., 2014). The inconsistencies between studies may be due to the different neuroimaging methods used to investigate tinnitus or heterogeneity of the tinnitus patients. Therefore, further studies are required to determine if the observed left hemispheric dominance is related specifically to tinnitus or some other factors.

### Limitations

Study limitations must be acknowledged. First, we admit that it is difficult to make direct causal inferences regarding the relationships between the brain functional network architecture and tinnitus characteristics in tinnitus patients, considering the cross-sectional nature of our experimental design and limited sample size. Further longitudinal studies involving a greater number of subjects are required. The very nature of BOLD-fMRI makes it difficult to measure resting-state network activity given that amplitude is not measured only correlations of BOLD signals (Logothetis et al., 2001). The relationship between the measured BOLD-fMRI signal and the underlying neural activity is still largely unknown. Further studies combining fMRI with EEG may help understand the exact relationship. Second, common criticisms regarding GCA algorithm for rs-fMRI data have been raised. For example, the fallacious effects might occur because of the systematic differences across brain regions in hemodynamic delays. GCA has been confounded by the presence of vascular anatomy (Webb et al., 2013). Additionally, changes in directionality could be caused by the differences in hemodynamic coupling in different regions (Smith et al., 2011). Furthermore, we attempted to exclude subjects with hyperacusis from our study because subjects with hyperacusis exhibited robust activation in selected brain regions, such as the primary auditory cortex, thalamus, and auditory midbrain (Gu et al., 2010). However, it would be useful to include subjects with hyperacusis in future studies so as to examine if intrinsic functional network architecture is disrupted in a similar manner to that was observed in our tinnitus patients without hyperacusis. Finally, although we attempt to reduce the MR scanner noise with earplugs, the subjects cannot be completely prevented from hearing some noise that probably alters the resting-state brain networks to varying degree (Logothetis et al., 2009). This confounding factor should be taken into consideration for all rs-fMRI studies related to the auditory systems.

## Conclusion

In this study, we show that disrupted functional network architecture within non-auditory regions revealed by DC and GCA are present in chronic tinnitus patients with normal hearing, without hyperacusis. Chronic tinnitus patients exhibited enhanced FC strength in bilateral SFG regions, which showed unidirectionally increased causal connectivity to frontal, motor, visual, somatosensory cortex and cerebellum. Furthermore, increased effective connectivity from bilateral SFG to OFC and SMA were positively correlated with tinnitus distress. Disturbance in brain functional network architecture will contribute to a better understanding of the neuropathophysiological mechanisms in tinnitus perception.

## Author Contributions

Y-CC and YF designed the experiment, collected the data, performed the analysis and wrote the manuscript. J-JX and C-NM collected the data. WX, JR, and XY contributed to the discussion and manuscript revision.

## Conflict of Interest Statement

The authors declare that the research was conducted in the absence of any commercial or financial relationships that could be construed as a potential conflict of interest.

## Footnotes

^1^http://www.fil.ion.ucl.ac.uk/spm

^2^http://www.restfmri.net

^3^https://afni.nimh.nih.gov/pub/dist/doc/manual/AlphaSim.pdf
